# Enhancing Paper’s Mechanical Strength and Antibacterial Properties through a Biopolymer-Based Coating

**DOI:** 10.1021/acs.iecr.5c01067

**Published:** 2025-06-06

**Authors:** Neha Sawant, Sara T. Caceres, Carin L. Garcia, Mario O. C. Lizardo, Carol Beaver, Santiago Aparicio, Mert Atilhan

**Affiliations:** † Department of Chemical and Paper Engineering, 4175 Western Michigan University, Kalamazoo, Michigan 49008, United States; ‡ Biology Department, Western Michigan University, Kalamazoo, Michigan 49008, United States; § Department of Chemistry, 16725 University of Burgos, Burgos 09001, Spain

## Abstract

This study presents a sustainable approach to improving the antibacterial and mechanical properties of paper through the application of a biopolymer-based coating derived from citric acid-modified soybean flour (SBFC) and carboxymethyl cellulose (CMC). The SBFC was synthesized via thermal cross-linking between citric acid and defatted soybean flour under optimized conditions (140 °C for 2 h, with Na_2_HPO_4_ as a catalyst), facilitating ester and amide bond formation that was confirmed via FTIR spectroscopy. Aqueous solutions of SBFC were subsequently blended with CMC at varying ratios, and a 50:50 formulation was optimized for the best performance of dispersion stability and coating performance. The final SBFC/CMC coating exhibited a 61.8% improvement in tensile strength compared to uncoated paper. Antibacterial activity was evaluated using Escherichia coli ATCC 25922 as a model Gram-negative bacterium, with bacterial reduction confirmed through CFU-based plate counts. The coating achieved a log_10_ reduction of 1.2–1.7 relative to that of the controls, corresponding to an 84–89% reduction in viable bacterial colonies. While testing was limited to a single bacterial strain, the results demonstrate promising antibacterial performance. This work highlights the potential of citric-acid-modified biopolymer coatings as multifunctional additives for improving the performance and hygiene properties of paper substrates, particularly in packaging applications.

## Graphical abstract

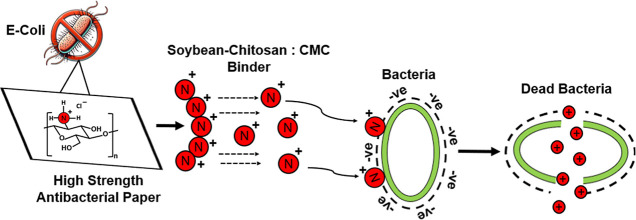

## 1 Introduction

The growing demand for paper-based packaging as a sustainable alternative to petroleum-derived plastics faces challenges due to paper’s natural hydrophilicity and lack of antimicrobial properties. To address these limitations, this study presents a simple, eco-friendly approach to enhance paper with antimicrobial and strength functionalities, making it suitable for diverse packaging applications.

Chitosan, a biocompatible and biodegradable polysaccharide derived from chitin, has emerged as a promising material for drug delivery and biomedical applications. Its unique properties, influenced by various extraction techniques, can be further enhanced through chemical modifications.^1^ The presence of reactive hydroxyl and amino groups facilitates intramolecular and intermolecular hydrogen bonding, contributing to chitosan’s nontoxic, antibacterial, antioxidative, and immunomodulatory properties. Cationic polysaccharides with amino or ammonium groups are valuable in various applications, and their chemical modification has been extensively studied to create anionic, cationic, and amphoteric materials for use in paper, textiles, and food industries.^2^ Chitosan shows a broad spectrum of antibacterial activity against both Gram-negative and Gram-positive bacteria, though its effectiveness varies across species.^3^ Chitosan’s antibacterial action is believed to result from a series of untargeted molecular events, including electrostatic interactions with teichoic acids on the bacterial cell wall, membrane disruption causing leakage of cellular components, interference with membrane-bound energy pathways, and potential stress responses due to local pH changes.^4^ Chitosan’s antimicrobial activity against Escherichia coli and S. aureus was investigated, finding that higher-molecular-weight chitosan forms a surface barrier blocking nutrient intake, while lower-molecular-weight chitosan permeates the cell, causing flocculation and physiological disruption; the former mechanism was seen better in S. aureus, than in E. coli, with 100% inhibition observed at a 1% chitosan concentration.^5^ A study showed an eco-friendly method for enhancing cellulose paper with antimicrobial and hydrophobic properties through chemical grafting of 3-aminopropyltriethoxysilane (APS) and cinnamaldehyde (CA), resulting in paper with long-term antibacterial and antifungal effects, good mechanical strength even when wet, excellent biocompatibility and biodegradability, and improved shelf life for strawberries, making it a promising material for fruit packaging.^6^ Biopolymer nanoparticles form a unique biobased latex emulsion that serves as an alternative binder to petrochemical-based systems in coated paper and paperboard manufacturing, with re-engineered starch nanoparticles that form a colloidal dispersion in water; these binders, when combined with titanium dioxide through reactive extrusion, enhance the optical properties of the coated paper by improving whiteness and brightness.

The imperative to mitigate the environmental burden associated with petroleum-based plastics has catalyzed significant research on sustainable packaging alternatives. Biopolymers, sourced from renewable biological origins, represent a highly promising class of materials owing to their inherent biodegradability, biocompatibility, and independence from fossil fuel feedstocks.^7^ Chitosan and soy protein, in conjunction with modified cellulosic materials, such as carboxymethyl cellulose (CMC), exemplify this potential. Soy protein, economically derived from abundant agricultural sources, exhibits favorable film-forming capabilities.^8–10^ Chitosan, the deacetylated derivative of chitin (a structural polysaccharide abundant in crustacean shells, insect exoskeletons, and fungal cell walls), offers unique antimicrobial properties alongside its biodegradability and nontoxicity.^11^

The antimicrobial action of chitosan is multifaceted yet fundamentally rooted in its polycationic character under acidic conditions. The positively charged amino groups (NH_3_^+^) present along the chitosan polymer chain engage in electrostatic interactions with negatively charged constituents of microbial cell membranes, such as lipopolysaccharides in Gram-negative bacteria and teichoic acids in Gram-positive bacteria.^12,13^ These interactions compromise membrane integrity, inducing leakage of intracellular components (proteins, nucleic acids, and ions), ultimately culminating in cell death.^14^ The degree of deacetylation (DDA) and molecular weight of chitosan are critical determinants of its antimicrobial efficacy. A higher DDA, reflecting a greater proportion of free amino groups, generally enhances antimicrobial potency due to increased positive charge density.^15^ The relationship between MW and antimicrobial activity is more nuanced; higher-molecular-weight chitosan may create a physical barrier impeding nutrient uptake, while lower-molecular-weight chitosan can penetrate the cell membrane, disrupting intracellular processes.^14^

Recent research has also emphasized the synergistic effects of combining chitosan with other plant-based proteins such as soy flour, highlighting improvements in film-forming capacity, water resistance, and antibacterial activity.^16,17^ Furthermore, soy protein has been studied extensively for its adhesive properties, and its functional groups can interact with chitosan to provide enhanced bonding and antimicrobial characteristics.^16^ Citric acid cross-linking is recognized as an effective, nontoxic strategy for stabilizing biopolymer-based coatings, as it can form ester linkages that improve thermal stability and moisture resistance.^17^ Additionally, blending chitosan with carboxymethyl cellulose (CMC) has been shown to strengthen the mechanical properties of coatings while retaining desirable antibacterial effects.^18^ In this study, citric acid was chosen as the modifying agent to optimize the reaction conditions and enhance antimicrobial activity. Citric acid was reacted with soy flour in the presence of sodium hypophosphite and then complexed with chitosan. A fixed amount of the modified soy flour additive, along with a 1% CMC solution slurry, was uniformly applied to hand sheets for all tests.^19^

## 2 Materials and Methods

### 2.1 Materials

OCC was provided by the Pilot plant. Table 1 gives a combined list of the chemicals used in this study. The bacterial strain was provided by the Biology Department at Western Michigan University.

**Table 1:** Chemicals Used in This Study

chemical	abbreviation	CAS No./CAS RN	lot	manufacturer
sodium chloride	NaCl	7647–14–5	Lot 243598	fisher chemical
agar technical solidifying agent	NA	9002–18–0	Lot 4256189	Difco TM
nutrient broth	NB		Lot 3763642	OXOID
citric acid, 99.5%		77–92–9	Source MKCT3065	Sigma-Aldrich
defatted soybean flour	SBF		2H4A1G106	KOSHER
sodium phosphate dibasic dihydrate	Na_2_HPO_4_	10028–24–7	126475	Fisher Scientific

The bacterial strain used for all antibacterial tests was E. coli ATCC 25922, a well-characterized standard strain obtained from the American Type Culture Collection. This strain is commonly employed in antimicrobial surface testing protocols due to its reproducibility and relevance in food safety and healthcare studies.

### 2.2 Modification and Optimization of the Defatted Soybean Flour

To increase its carboxylic content, defatted soybean flour was chemically modified by esterification with citric acid. In this study, varying concentrations of citric acid (20%, 40%, 60%, 80%, and 100%) were used, calculated based on the weight of the soybean flour. For clarity throughout the paper, each formulation is labeled as C*x*S*y*, where C and S represent the gram amounts of citric acid and soybean flour, respectively. For example, C_3_S_5_ corresponds to 3 g of citric acid and 5 g of soybean flour.

Citric acid was dissolved in a minimal amount of deionized water, and sodium hypophosphite (SHP)/Na_2_HPO_4_ was added as a catalyst. The solution was stirred thoroughly using a glass rod until homogeneous. Defatted soybean flour was then added to the citric acid-catalyst solution with continuous stirring to ensure an even distribution of reactants. The mixture was kept in an air oven for the desired time and temperature.

### 2.3 Complexation of Optimized Modified Soybean Flour with Chitosan

The soybean–chitosan complex preparation began by dispersing 1 g of citric acid-modified soybean flour (previously prepared under optimized conditions of 140 °C, 100% citric acid, 2 h, and 15% Na_2_HPO_4_) in 20 mL of deionized (DI) water in a beaker. In a separate beaker, 1 g of chitosan was dissolved by adding it to a solution prepared with 1 g of HCl dissolved in 79 mL of DI water. Medium-molecular-weight chitosan was used. The reaction temperature was chosen based on established literature demonstrating that temperatures above 100 °C promote the thermal dehydration of citric acid into reactive cyclic anhydride intermediates.^20,21^ These anhydrides exhibit significantly enhanced reactivity toward nucleophilic functional groups such as hydroxyl and amine groups on soy protein chains, enabling efficient ester and amide bond formation. Although the initial reaction occurs in an aqueous environment, the system progressively transitions into a solid state as moisture evaporates during thermal curing, which further facilitates the cross-linking reaction. Since chitosan requires an acidic environment for solubilization, the acidic medium facilitates complete dissolution. The chitosan solution was stirred until it was fully homogeneous. The two solutions were then combined in a conical flask and heated at 80 °C for 90 min under continuous stirring to promote complexation. This procedure yielded a 50:50 (w/w) soybean-flour–chitosan complex (C5S5).

Upon heating at the optimized temperature of 140 °C, citric acid undergoes dehydration, forming a reactive cyclic anhydride intermediate

$$citric\;acid\overset{\Delta H}{\to }cyclic\;anhydride+H_{2}O$$

This cyclic anhydride intermediate can readily react with nucleophilic functional groups in soy protein, specifically hydroxyl (−OH) groups, such as those present in serine or threonine residues, to form ester linkages. Esterification reaction

protein−OH+cyclicanhydride→protein−O−C(O)−(citricacidresidue)

Additionally, amino (−NH_2_) groups, such as those from lysine residues or N-terminal groups, form amide bonds with the anhydride intermediate. Amide formation reaction

$$protein-{NH}_{2}-cyclic\;anhydride\to protein-NH-C\left(O\right)-\left(citric\;acid\;residue\right)$$

These reactions facilitate extensive covalent cross-linking within the soy protein structure, significantly enhancing the mechanical properties and water resistance of the modified soybean flour.^22–24^

### 2.4 Characterization

#### 2.4.1 FTIR Analysis

Fourier transform infrared (FTIR) spectroscopy was used to characterize the functional groups in soybean flour before and after modification. The analysis was conducted using a PerkinElmer Spectrum 100 FTIR spectrophotometer with an attenuated total reflectance (ATR) accessory.^25^ Spectra were recorded in the range of 4000–600 cm^–1^ with background correction performed before each measurement to eliminate atmospheric interference.^26^ A small amount of soybean flour and modified soybean flour was placed individually on the ATR crystal, and uniform pressure was applied using the built-in pressure clamp to ensure proper contact. The FTIR spectra, collected in absorbance mode, provided details on structural modifications such as the esterification reaction. This analysis was essential for evaluating changes in functional groups, to identify and confirm the effectiveness of the modification process.^27,28^

#### 2.4.2 Titration Procedure

The carboxylic content of the modified soybean flour was determined using an acid–base titration. 0.5 g of each soybean flour sample was weighed and transferred into 250 mL Erlenmeyer flasks. 20 mL of a 0.1N NaOH solution was added to each flask using a pipet. The flasks were placed on a shaker and mixed for 30 min to allow thorough dispersion of the flour samples in the NaOH solution. Figure 1 shows the sample preparation for titration.^28^

**Figure 1:**
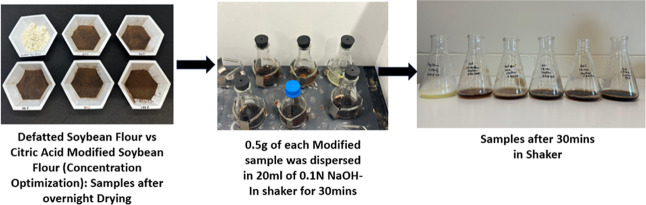
Sample preparation for titration.

After mixing, the flasks were removed from the shaker, and 3 drops of phenolphthalein indicator were added to each flask. The solution should turn pink, indicating a basic medium. Next, a buret was filled with a 0.1 N HCl solution, and the initial buret reading was recorded. Titration was begun by placing one flask under the buret and slowly adding HCl while continuously swirling the flask in a circular motion to mix the contents. HCl was continued to be added until the pink color disappeared, reaching the end point. The final buret reading was recorded, and then the volume of HCl used was calculated by subtracting the initial reading from the final reading. The carboxyl content, expressed in milliequivalents per 100 g (meq/100 g), was calculated using the formula^28^

$$carboxyl\;content\;\left(meq/100\;g\right)=\frac{\left(V_{2}-V_{1}\right)\times N\times 100}{W}$$

where *N* = normality of HCl, *V*_2_ = volume of HCl without the sample, *V*_1_ = volume of HCl with the sample, and *W* = weight of the sample.

#### 2.4.3 Scanning Electron Microscopy

A scanning electron microscope (SEM) operates similarly to an optical microscope but uses a focused beam of electrons instead of light to capture images and analyze a specimen’s structure and composition. In this study, the surface morphology of the control handsheet, SBFC/CMC-coated handsheets, and SBFC-coated new sheets was analyzed by using a JEOL JSM-IT200 scanning electron microscope (SEM). To enhance the conductivity and improve image contrast, a thin layer of gold was sputter-coated onto the samples under vacuum conditions using a DII-29010SCTR Smart Coater before imaging. The electron source generates a beam that is accelerated toward the specimen by using a positive electrical potential. As the electrons interact with the atoms in the sample, they generate signals that provide insight into surface topography, composition, and electrical properties. These signals are then processed and converted into detailed images of the specimen.^28,29^

#### 2.4.4 Handsheet Preparation and Coating

OCC fibers were obtained from the Pilot plant at Western Michigan University. The preparation of handsheets was performed according to TAPPI Standard-TAPPI T220. Initially, a 0.48% fiber slurry was prepared by using oven-dried fibers. The fibers were thoroughly dispersed in water to create a homogeneous mixture. This slurry was used as the base for all of the handsheets. The handsheets were then formed by using a TAPPI Standard handsheet mold. The target dry weight of each handsheet was about 1.2 g. Following this, the wet handsheets were carefully removed from the mold and pressed between blotting papers for 5 min, followed by a second press to remove excess water. This step was crucial for achieving a uniform sheet thickness. This process is presented in Figure 2. They were left to dry overnight in the paper testing room. This helps make sure the base hand sheets are completely dry before applying the coating. A 50:50 SBFC/CMC vol/vol binder solution was prepared. The handsheets were coated with the binder solution using a 6 MIL wet film. The coating should be spread consistently on the sheets. After coating, a hair dryer was used to dry the handsheets. The dryer speeds up the drying process and ensures that the binder sticks properly. Once dried, the coated sheets were used for further testing.

**Figure 2:**
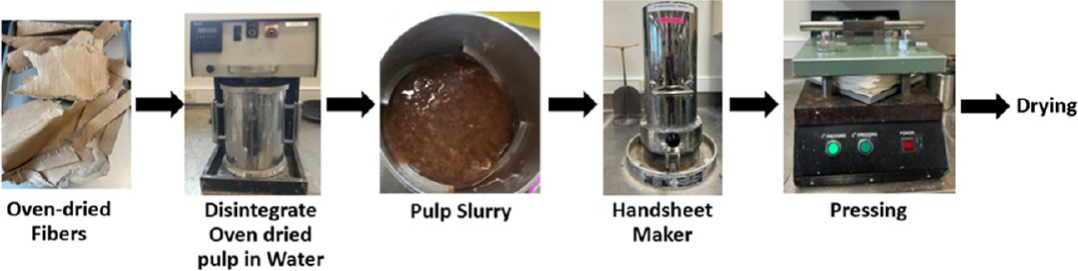
Lab-scale handsheet-making procedure.

#### 2.4.5 Soybean Flour-Chitosan and CMC Binder (SBFC/CMC)

The soybean flour-chitosan additive and CMC binder (SBFC/CMC) were prepared by first preparing the modified soybean-chitosan slurry. In another conical flask, a 1% carboxymethyl cellulose (CMC) solution was prepared by adding CMC to DI water and stirring it at room temperature until a homogeneous solution was formed. This CMC solution was mixed with the soybean–chitosan complex in varying ratios (30%:70%, 20%:80%, and 40%:60%) to create the SBFC/CMC mixture. 30%:70% and 20%:80% mixtures solidified after mixing. The 40%:60% mixture showed slight agglomeration, but no significant issues were observed. These solutions were then utilized as coating solutions on OCC handsheets and used for further tests (Figure 3).

**Figure 3:**
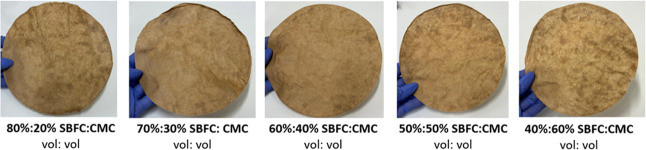
Coated handsheets with the SBFC/CMC solution in varying ratios.

#### 2.4.6 Tensile Strength Testing of Paper-TAPPI T494

Tensile strength testing was conducted to compare the performance of control handsheets and SBFC/CMC-coated handsheets, using the TAPPI T494 standard. Ten strips of each sheet were tested for the tensile test. Visual representation of the tensile strength can be seen in Figure 4.

**Figure 4:**
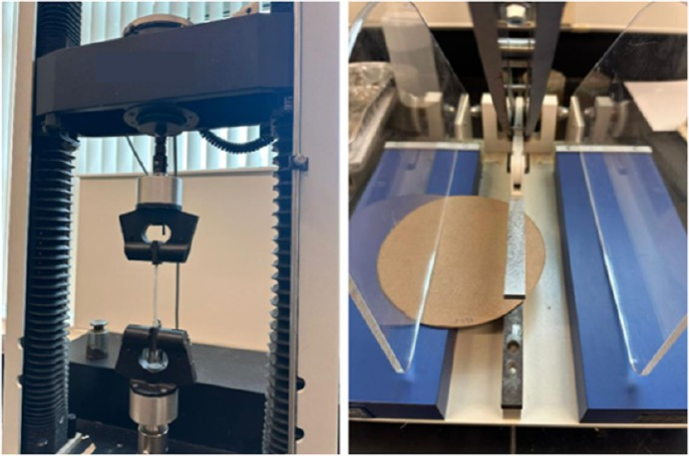
Tensile strength measurement test setup.

#### 2.4.7 Dynamic Viscosity Measurement of the 50:50 SBFC/CMC Solution

The dynamic viscosity of the 50:50 SBFC/CMC (vol: vol) solution was measured using an Anton Paar ViscoQC300 instrument (Figure 5).

**Figure 5:**
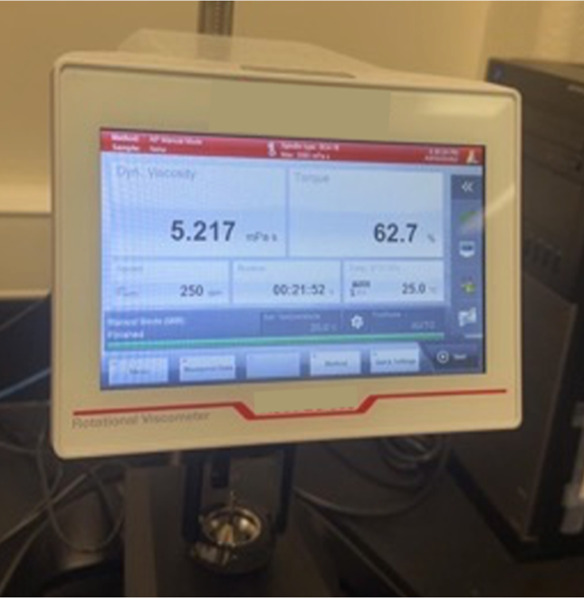
Anton Paar ViscoQC300 instrument.

### 2.5 Antibacterial Test-FTTS-FA-002

The antimicrobial test was performed according to FTTS-FA-002, using E. coli as the bacterial strain provided by the Biology Department at Western Michigan University. The test began by preparing the bacterial culture from the initial colony, using a disposable inoculum loop to transfer bacteria into the nutrient broth (NB) and incubating for 18–24 h to obtain the required bacterial concentration of (1–2) × 10^8^ CFU/mL. The OD was adjusted at 600 nm using a UV–vis spectrophotometer. Following this, the bacterial culture was further incubated in NB for 2 h at 37 °C ± 2 °C with shaking to reach a target bacterial concentration of 10^7^ CFU/ml, and a 20-times diluted solution was prepared for testing (Figure 6).

**Figure 6:**
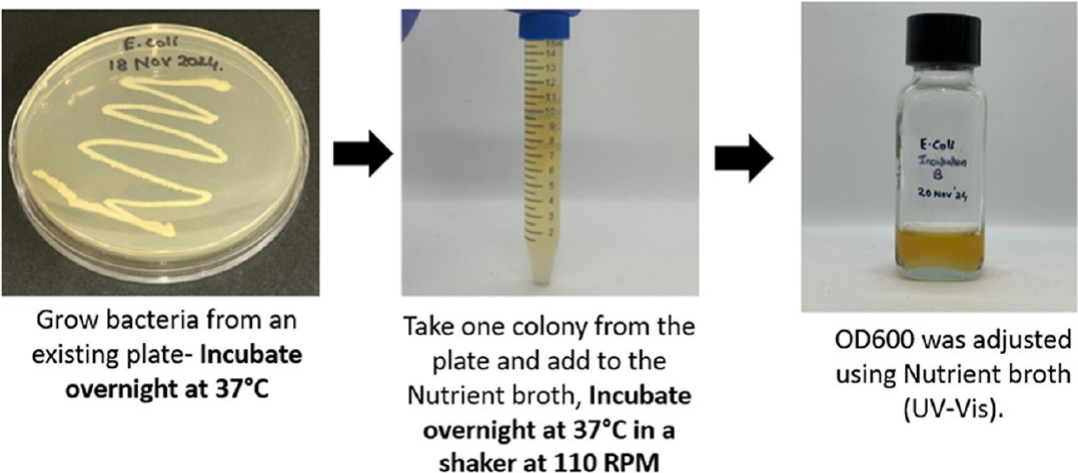
Preparing the E. coli bacterial culture.

#### 2.5.1 Immediate Incubation after Shakeout

Circular swatches of the test specimen (4.8 ± 0.1 cm) were cut from the sample, and 1 mL of the diluted bacterial culture was applied to the specimen and untreated control swatches. Each swatch was then treated with 100 mL of a neutralizing solution (8.5 g sodium chloride in 1000 mL of distilled water), followed by vortex mixing to shake out the bacteria immediately (Figure 7). Serial dilutions of 10^1^, 10^2^, 10^3^, and 10^4^ were made from the bacterial solution, and 100 μL of each dilution was pipetted onto a Petri dish with solidified sterile Nutrient Agar. The spread plate method was used. The plates were observed at 37°C ± 1 °C for 24–48 h for bacterial growth and analyzed to determine the antimicrobial effectiveness of the sample.

**Figure 7:**
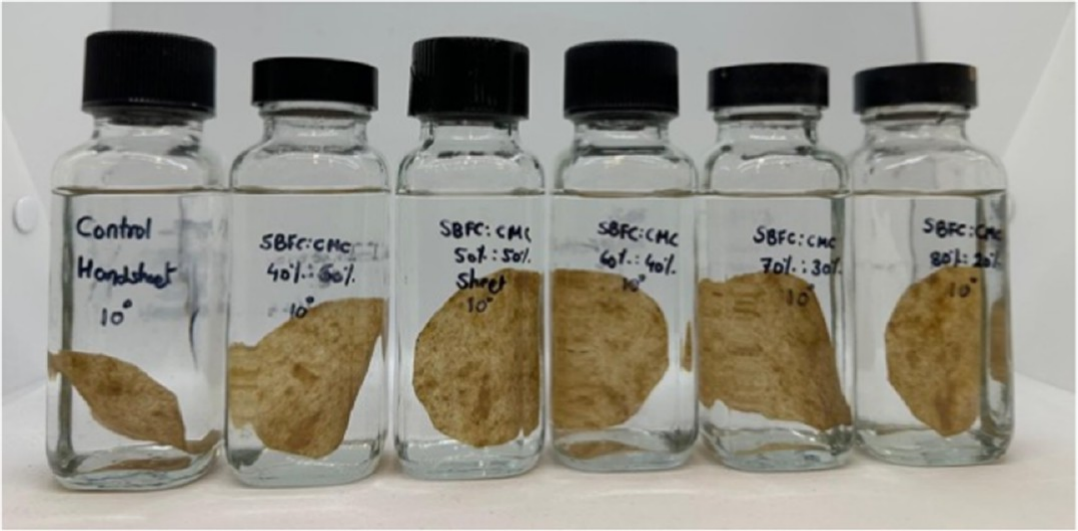
Circular swatches of handsheets with bacteria in 100 mL of the neutralizing solution.

Each treatment was tested in triplicate biological replicates. For each replicate, serial dilutions of bacterial suspensions were plated in duplicate, and only plates yielding between 30 and 300 colonies were used for CFU/mL calculations, consistent with ISO 22196:2011 guidelines (Maitz et al., 2024).^30^

## 3 Results and Discussions

The esterification reaction for modifying defatted soybean flour with citric acid was carried out at 120 °C for 2 h. The reaction time was carefully monitored and started only after the mixture reached the desired temperature. Following the completion of the reaction period, images of the samples were taken to document visual differences in color and appearance, confirmed with varying concentrations of citric acid used (20%, 40%, 60%, 80%, and 100%); Figure 8. Each formulation is coded as C*x*S*y* to indicate the specific citric acid-to-soybean flour ratio used during synthesis.

**Figure 8:**
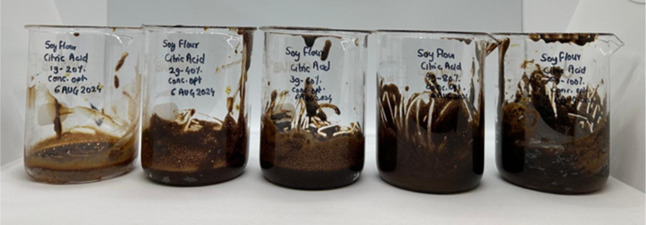
Effect of citric acid concentration on soybean flour (before the washing step).

These pictures indicate the effect of the citric acid concentration on the modification process before the washing and purification steps.

After each reaction, the modified soybean flour was cooled and centrifuged for multiple cycles (7–15 cycles) to remove unreacted citric acid. Deionized water was used for washing between centrifuge cycles to ensure thorough purification. The washed product was dried overnight in an air oven before analysis (Figure 9). pH was recorded after the final wash for each modification and is presented in Table 2.

**Figure 9:**
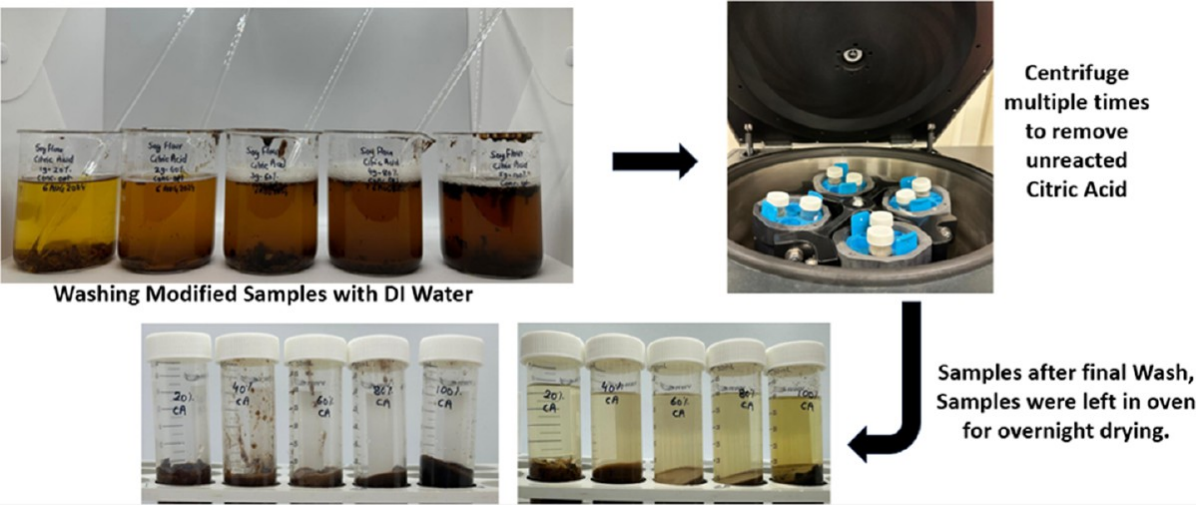
Washing of citric acid-modified soybean flour via centrifugation.

**Table 2:** pH after the Final Wash: Concentration Optimization of Soybean Flour

citric acid concentration	citric acid (g)	defatted soybean flour (g)	formulation code C*x*S*y* ^a^	Na_2_HPO_4_–15% (g)	pH after final wash
20%	1	5	C1S5	0.15	3.78
40%	2	5	C2S5	0.3	3.37
60%	3	5	C3S5	0.45	3.35
80%	4	5	C4S5	0.6	3.34
100%	5	5	C5S5	0.75	3.31

a C*x*S*y*: C represents the weight (in grams) of citric acid, and S represents the weight (in grams) of soybean flour used in each formulation. Note: Estimated pH values for 5:0 and 0:5 formulations were obtained from the literature: citric acid (∼0.1 M) ≈ pH 2.1,^31^ defatted soybean flour slurry ≈ pH 6.7.^32^

pH after the final washing step for concentration optimization is given in Table 2.

Similarly, the reaction time and temperature were optimized by conducting trials at five temperatures (90 °C, 100 °C, 110 °C, 120 °C, and 130 °C) for various durations (0.5, 1, 1.5, 2, and 2.5 h). The visual effects of reaction time on the citric acid modification of soybean flour prior to washing are illustrated in Figure 10, highlighting the progressive changes in color and consistency associated with extended thermal exposure.

**Figure 10:**
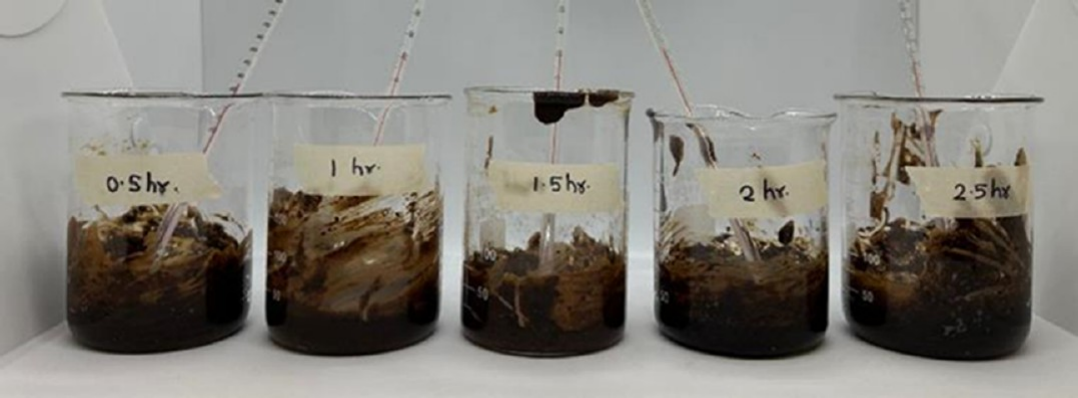
Effect of time on soybean flour (before washing step).

The reaction was followed by a washing step (Figure 11).

**Figure 11:**
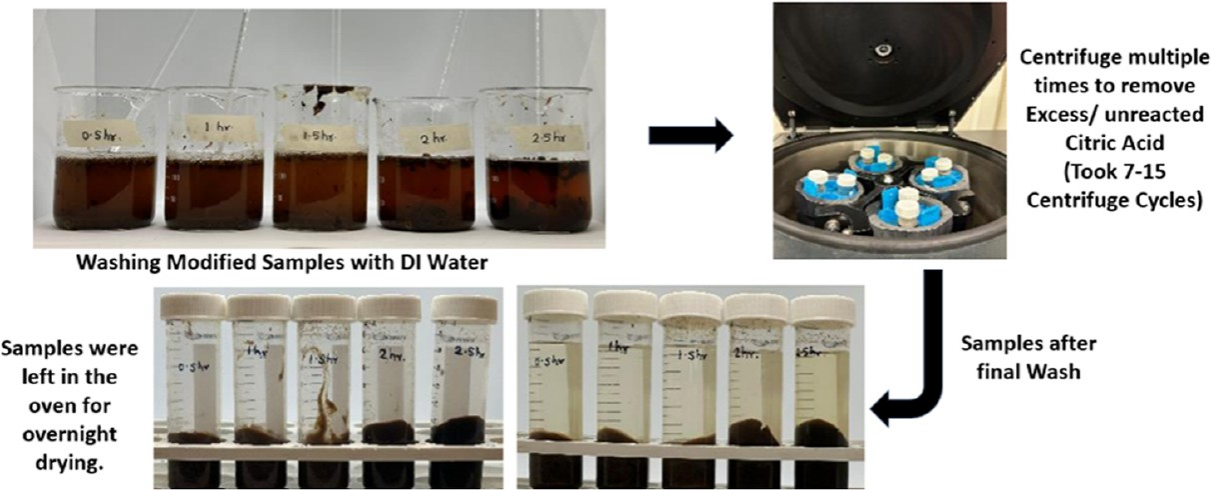
Time optimization: washing of citric acid-modified soybean flour via centrifugation.

pH after the final washing step for time optimization is presented in Table 3.

**Table 3:** pH after the Final Wash: Time Optimization of Soybean Flour

reaction time (hr.)	formulation code C*x*S*y*	Na2HPO_4_–15% (g)	pH after the final wash
0.5	C5S5	0.75	3.17
1.0	C5S5	0.75	3.11
1.5	C5S5	0.75	3.14
2.0	C5S5	0.75	3.21
2.5	C5S5	0.75	3.28

The effect of the reaction temperature on the modification process before the washing and purification steps can be seen in Figure 12.

**Figure 12:**
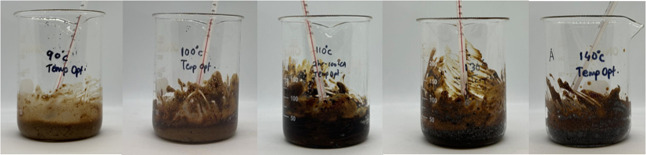
Effect of temperature on soybean flour (before the washing step).

The reaction was followed by a washing step (Figure 13).

**Figure 13:**
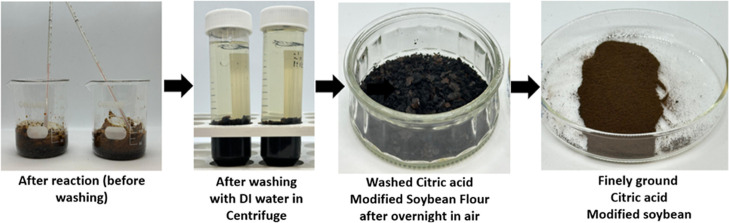
Final optimized modified soybean flour (140 °C, 100% CA, and 2 h, with 15% Na_2_HPO_4_).

The goal of optimizing the citric acid (CA) modification of soybean flour was to find a balance between the high carboxyl content and efficient processing conditions. In early tests, we used extreme conditions of 160 °C for 2.5 h with 100% CA, which gave a carboxyl content of 385.229 mequiv/100 g. Hence, we decided on final conditions of 140 °C, 100% CA, and 2 h, with 15% Na_2_HPO_4_ (Figure 14). This gave a similar carboxyl content of 383.68 mequiv/100 g. The small difference of only 1.55 mequiv/100g made the lower temperature and slightly lower time an efficient choice. A summary of the carboxylic acid content and the effect of concentration, reaction time, and reaction temperature is given in Figure 14.

**Figure 14:**
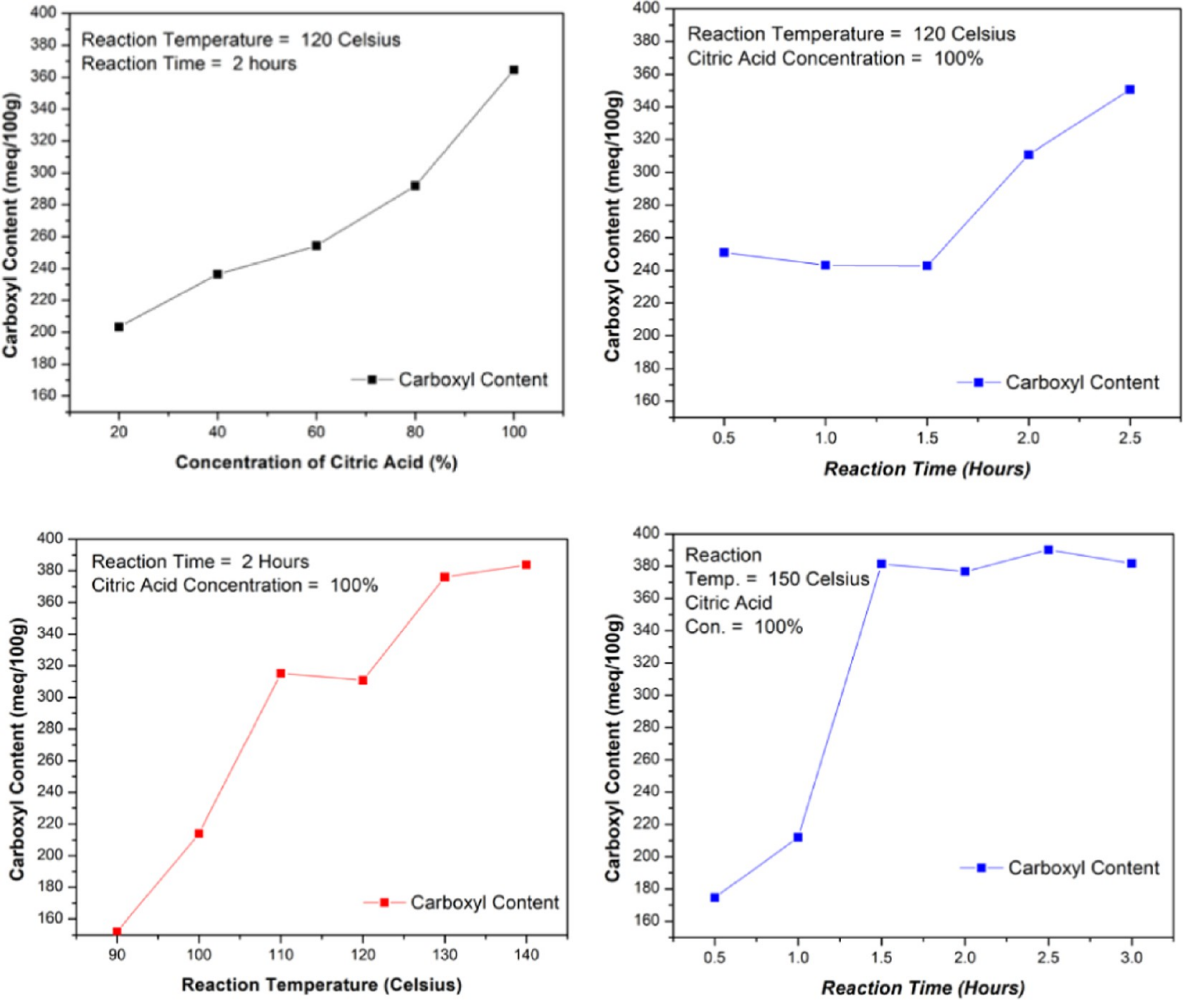
COOH^–^ concentration, time, and temperature optimizations.

The 50:50 citric acid to soybean flour ratio was selected after evaluating several formulations (30:70 to 60:40) by UV–vis spectroscopy. The 50:50 blend produced the highest absorbance in the 300–400 nm range, corresponding to optimal cross-linking intensity. The 50:50 ratio offered the best balance between cross-linking reactivity and formulation stability.

### 3.1 Scanning Electron Microscopy (SEM)

The optimized soybean flour was analyzed with SEM and FTIR spectroscopy. The surface of soybean flour went through significant changes after modification with citric acid at 140 °C for 2 h, as observed through SEM. The SEM images (Figure 15) showed that the untreated soybean flour initially had a smooth and uniform surface morphology. Green dashed circles highlight areas of increased surface roughness observed in the citric acid–modified soybean flour, while yellow dashed circles indicate regions where particles appear more compact and fused, suggesting enhanced structural cohesion resulting from cross-linking.

**Figure 15:**
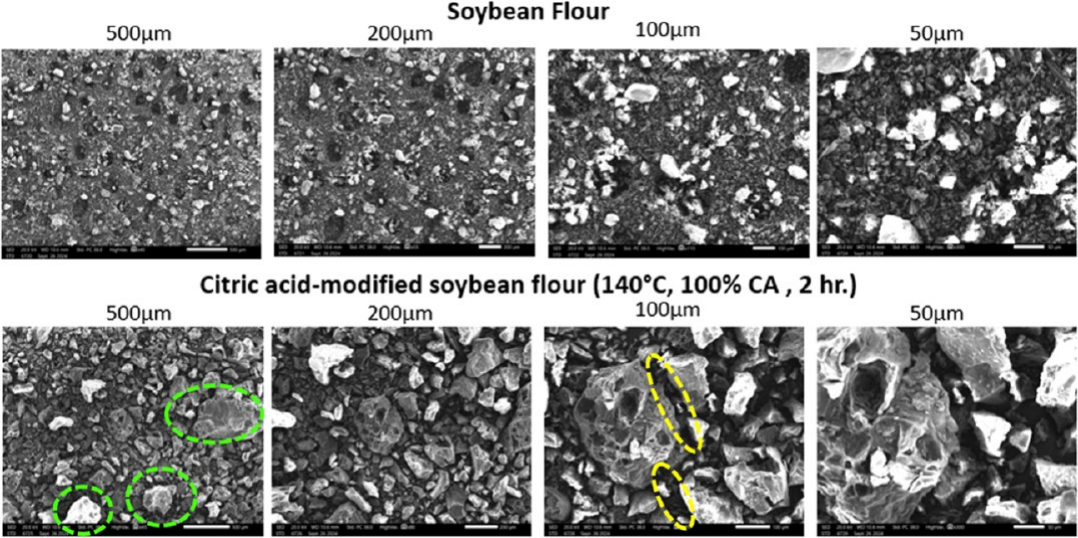
SEM of soybean flour vs citric acid-modified flour.

However, after modification, the surface became rougher and coarser. This could be due to the esterification reaction between the hydroxyl groups of the soybean flour and the carboxylic groups of citric acid, which led to an increased carboxylic content. The chemical modification process could have added to the structural changes in the flour particles; this could be due to cross-linking and degradation due to the high temperature and long reaction time. Hence, these changes suggest successful functionalization.

### 3.2 SEM: Comparison of Respective Control Sheets with Those of SBFC/CMC-Coated Sheets and Only SBFC-Coated New Sheets

After coating control handsheets with SBFC/CMC, scanning electron microscopy (SEM) analysis shows significant changes in surface morphology. The SEM images show that the coating material effectively fills the gaps and voids between the fibers, leading to a denser network (Figure 16). These images reveal a significant difference in surface morphology between the control and the coated handsheets. In the control sample, green-labeled regions highlight prominent voids and exposed fibers, indicating an unfilled porous structure. In contrast, the yellow-shaded areas in the coated handsheet illustrate regions where interfiber spaces have been successfully filled with the SBFC/CMC binder, resulting in a denser, more integrated fiber network.

**Figure 16:**
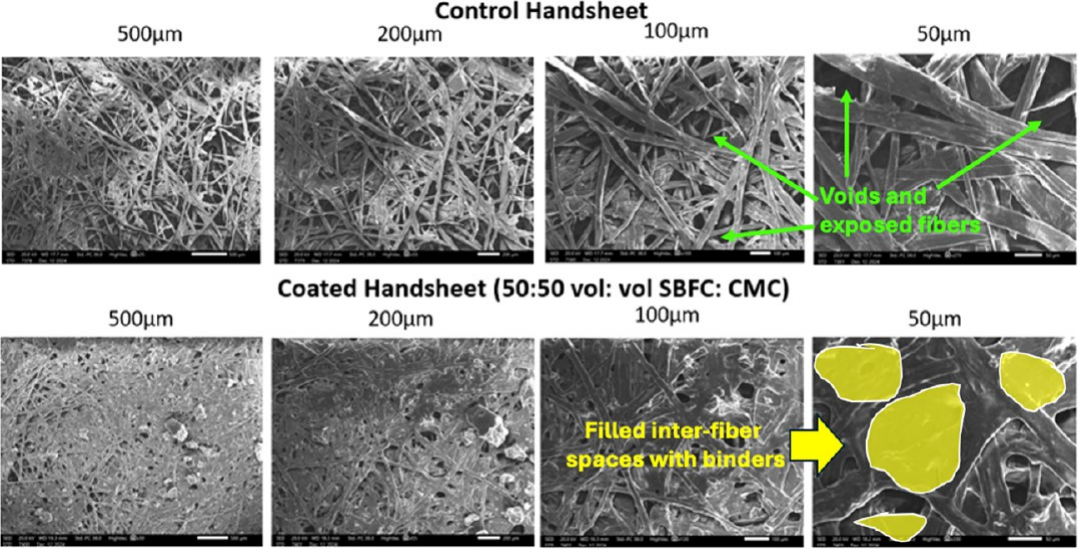
SEM of control handsheets vs 50:50 vol: vol SBFC/CMC-coated handsheets.

Surprisingly, SBFC alone was enough to coat the new sheet (Figure 17). The contrast between coated and uncoated samples shows the role of the applied coating in modifying the paper and enhancing its strength and antibacterial properties.

**Figure 17:**
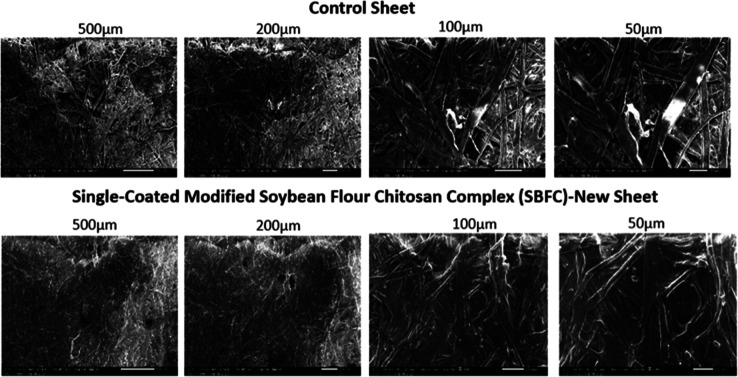
SEM of the new sheet coated with the SBFC solution.

### 3.3 FTIR Spectroscopy

The FTIR spectra of unmodified soybean flour and citric acid-modified soybean flour show changes from the esterification reaction (Figure 18). The unmodified soybean flour showed peaks at 3273, 1635, 1531, 1393, 1237, 1047, and 998 cm^–1^. The peak at 1635 cm^–1^ indicates C=O stretching of amide bonds, which is a prominent indicator of proteins.^33^ The peak at 1531 cm^–1^ represents N–H bending and C–N stretching vibrations.^34^ The peaks at 1237 and 1047 cm^–1^ are due to the C–O stretching in carbohydrates, and the peak at 998 cm^–1^ corresponds to C–H bending.

**Figure 18:**
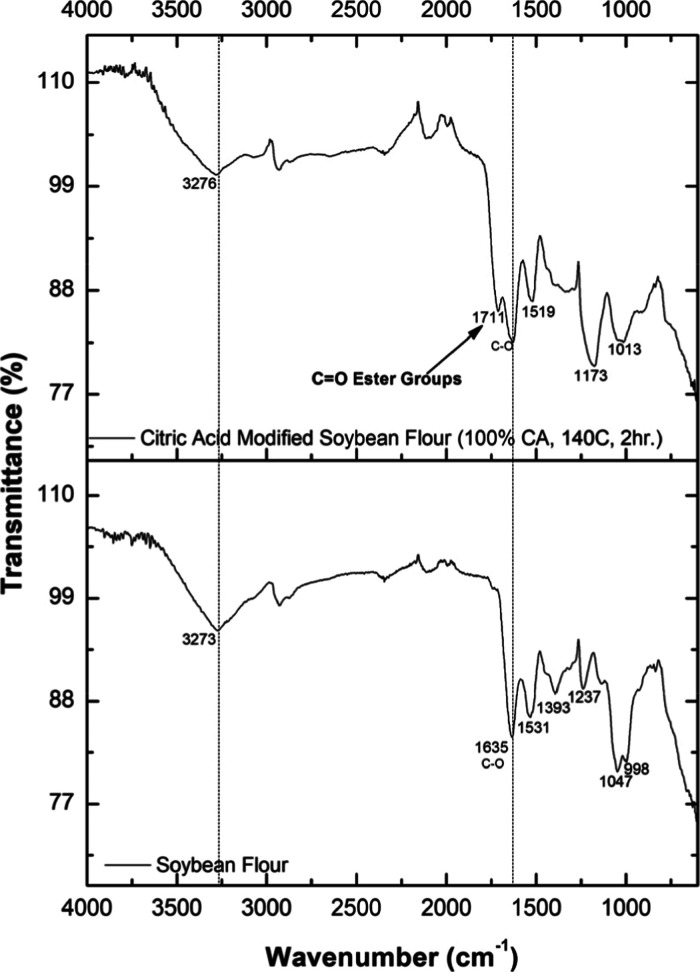
FTIR spectra of unmodified soybean flour and citric acid-modified soybean flour.

In formulation C5S5 (5g of citric acid and 5 g of soybean), a new peak at 1711 cm^–1^ (marked in Figure 18) indicates the presence of ester carbonyl (C=O) groups formed during the esterification process with citric acid. Additionally, the peak at 1519 cm^–1^, corresponding to N–H bending and C–N stretching, indicates the intact protein structures. The appearance of peaks at 1173 and 1013 cm^–1^ is due to C–O–C and C–O stretching vibrations from ester linkages.^35^

### 3.4 SBFC/CMC Binder

To prepare the final coating solution, the citric-acid-modified soybean flour complex (SBFC) was blended with a carboxymethyl cellulose (CMC) solution to form a hybrid binder. A 1% (w/v) CMC stock solution was first prepared by dispersing CMC powder in deionized water and heating it to 80 °C under continuous stirring at 500 rpm for 30 min to ensure complete dissolution and homogeneity. In parallel, the SBFC complex that was obtained after the optimized thermal reaction and purification steps was redispersed in deionized water to match the viscosity and volume of the CMC solution. The two components were then combined in varying volume-to-volume ratios, 30:70, 20:80, 40:60, and 50:50 (SBFC/CMC), and stirred at room temperature for 15 min at 500 rpm to promote uniform mixing.

Formulation stability was visually and physically assessed immediately after mixing. Both the 20:80 and 30:70 SBFC/CMC formulations exhibited premature solidification and poor flow behavior, likely due to excessive hydration of the CMC matrix, overpowering the dispersed SBFC particles. The 40:60 ratio produced a workable mixture but exhibited minor agglomeration and phase separation over time. In contrast, the 50:50 mixture yielded a homogeneous, stable binder with consistent flow behavior and good coating spreadability performance, making it ideal for application to handsheets and subsequent testing. As a result, this optimized ratio was selected for all antibacterial, mechanical, and morphological evaluations (Figure 19). Other ratios were excluded from further testing due to formulation instability, inadequate coating behavior, or poor mechanical film integrity, making them impractical for systematic evaluation and comparative analysis.

**Figure 19:**
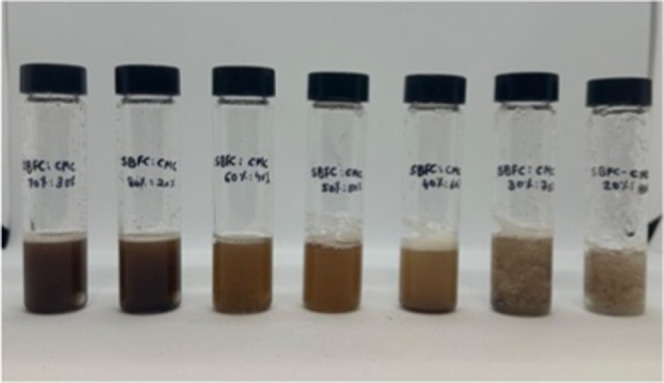
Solutions of varying SBFC/CMC ratios (vol: vol).

### 3.5 Strength Test

The control handsheets showed an average tensile strength of 0.959 kgf, while the SBFC/CMC-coated handsheets, with a 50:50 ratio of SBFC to CMC, showed a significantly higher tensile strength of 1.552 kgf (Table 4). The SBFC/CMC-coated handsheets showed a 61.83% improvement in tensile strength compared to the control handsheets. This increase in tensile strength indicates that the SBFC/CMC coating could be due to the properties of the modified soybean flour and chitosan binder (Table 4), providing improved strength and resistance to applied force. These results suggest that the SBFC/CMC coating could be used to improve the mechanical properties of paper-based materials.

**Table 4:** Tensile Strength of Control vs SBFC/CMC-Coated Handsheets

average control sheet weight was 1.18–1.2 g					
tensile strength (kgf)					
Sr. No	control handsheet	coated handsheet (50:50 SBFC/CMC)	Sr. No	control handsheet	coated handsheet (50:50 SBFC/CMC)
1	1.195	1.522	6	0.8429	2.059
2	0.7785	1.141	7	0.9664	1.35
3	1.254	1.608	8	0.7544	1.723
4	0.9315	1.522	9	1.025	1.713
5	0.8725	1.412	10	0.9745	1.474

Figure 20 presents the tensile strength comparison between the uncoated control and SBFC/CMC-coated handsheets, showing a marked increase in mechanical performance attributed to the binder application.

**Figure 20:**
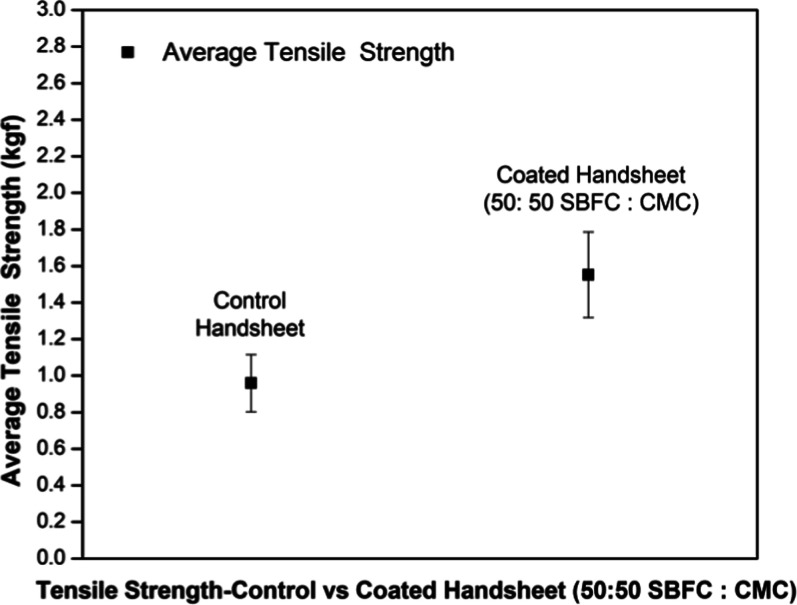
Comparison of tensile strength of control vs SBFC/CMC-coated handsheets.

The bar graph illustrates a clear increase in the average tensile strength for the SBFC/CMC-coated handsheets compared to the control, with error bars confirming the reproducibility of the measurements. This supports the effectiveness of the coating in reinforcing the paper structure.

### 3.6 Dynamic Viscosity Measurement of the 50:50 SBFC/CMC Solution

The dynamic viscosity of the 50:50 SBFC/CMC (vol: vol) solution was measured using an Anton Paar ViscoQC300 instrument, with the viscosity recorded as 5.217 mPa·s. This relatively low viscosity indicates that the solution was not highly viscous. Additionally, during the measurement, the solution required manual stopping, and it probably may have started to lose its homogeneity over time. This could suggest that the components of the solution began to separate or form inconsistencies, affecting the viscosity.

### 3.7 Antibacterial Test Results

In trial 1, the OD was measured at a fixed wavelength of 600 nm; the OD600 was adjusted to 0.104. The bacterial colony count for the control E. coli sample was 5, while the control handsheet had a slightly reduced count of 4, resulting in a 20% reduction in bacterial growth. The coated handsheet (50:50 SBFC/CMC) showed a significantly lower colony count of 1, with a 75% reduction compared to the control handsheet and an 80% reduction compared to the control E. coli sample (Figures 21 and 22).

**Figure 21:**
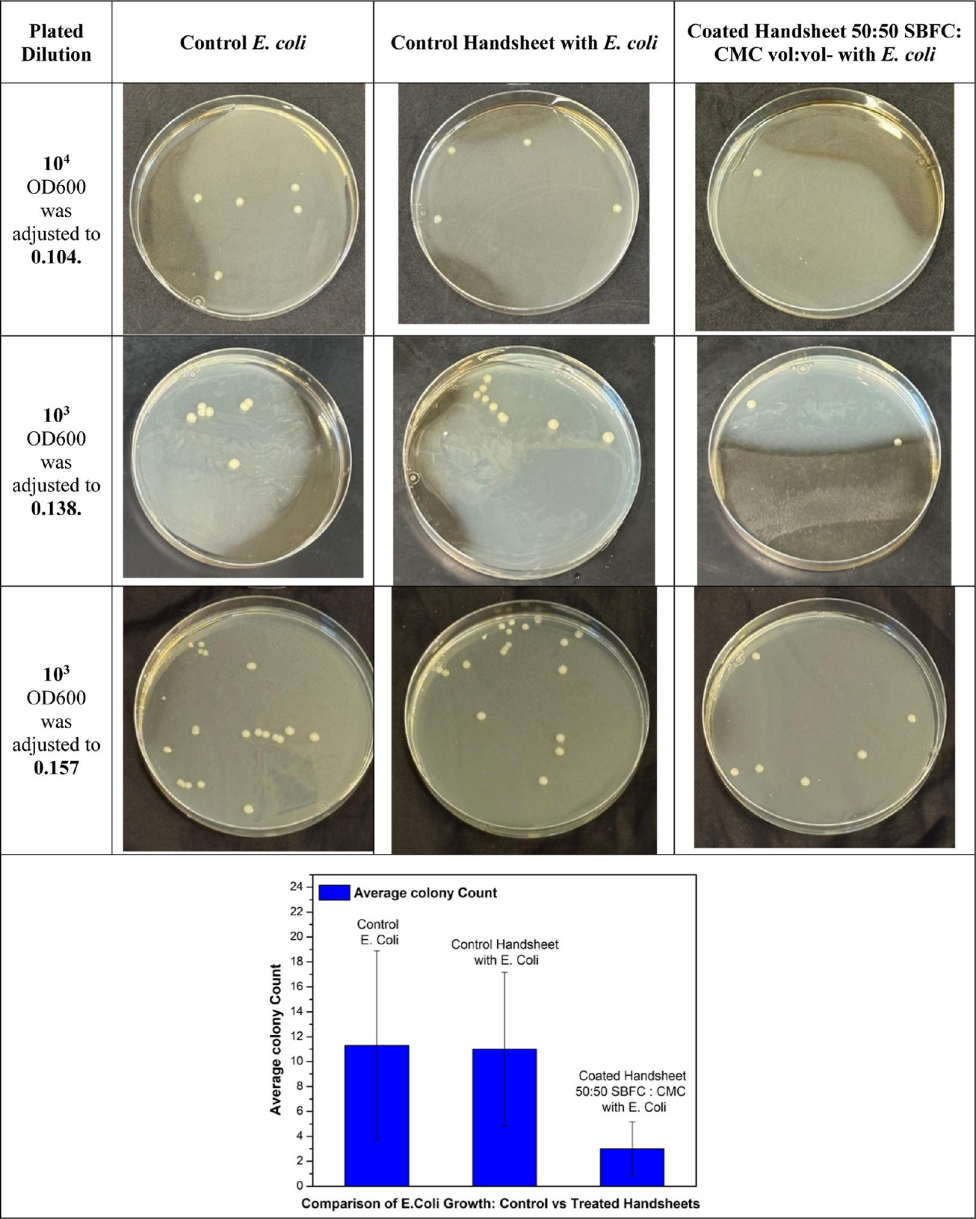
Antibacterial test results for coated handsheet 50:50 SBFC/CMC vol:vol, with E. coli.

**Figure 22:**
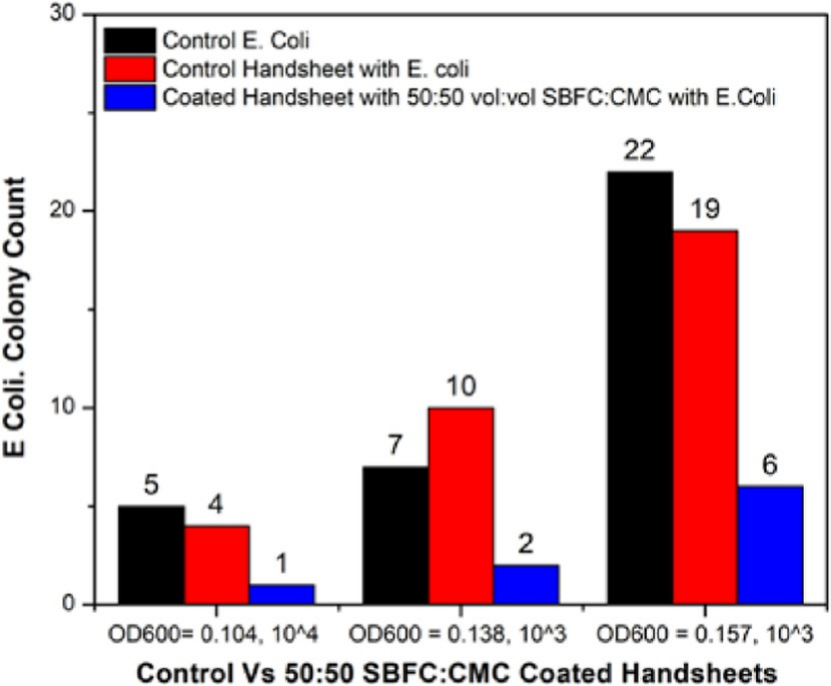
E. coli bacterial counts for control vs 50:50 SBFC/CMC-treated sheets.

In trial 2, the OD was measured at a fixed wavelength of 600 nm; the OD600 was adjusted to 0.138. The control E. coli sample had a colony count of 7, but the control handsheet showed an increase to 10 colonies, likely due to variability in handling or environmental factors. Despite this increase, the coated handsheet reduced the colony count to 2. Compared to the control handsheet, the coated handsheet achieved an 80% reduction, and compared to control E. coli, the reduction was 71.43% (Figures 21 and 22). The bar graph in Figure 21 presents the average E. coli colony counts following exposure to control and SBFC/CMC-coated handsheets, with standard deviation error bars representing variation across three independent replicates. While the error bars are relatively large, this reflects variability inherent to bacterial adhesion, growth kinetics, and handling during plating; all replicates consistently demonstrated a reduction in viable colonies for the coated handsheets. The reproducible trend supports the antibacterial potential of the coating.

In trial 3, the OD was measured at a fixed wavelength of 600 nm; the OD600 was adjusted to 0.157. The control E. coli sample showed a high colony count of 22, while the control handsheet had a slightly lower count of 19, representing a 13.64% reduction. The coated handsheet, however, significantly reduced the colony count to 6. This represents a 68.42% reduction compared to the control handsheet and a 72.73% reduction compared to that of control E. coli. The consistent performance of the coated handsheet across all trials demonstrates its effectiveness in minimizing bacterial growth. Overall, the coated handsheet (50:50 SBFC/CMC) was better than both the control handsheet and the control E. coli handsheet in each trial. The reduction in bacterial colonies indicates the effectiveness of the coating and results in antibacterial properties (Figures 21 and 22).

To ensure methodological rigor and reproducibility, all conditions were tested in three independent biological replicates with technical duplicates. CFU counts were normalized to the original inoculum volume and expressed as CFU/mL. Antibacterial effectiveness was quantified using log_10_ reduction relative to control samples, consistent with established protocols in antimicrobial surface assessments.^36,37^

To align with standard quantitative microbiological evaluation practices, bacterial reduction was recalculated and expressed in terms of log10 reduction. Across three trials, 50:50 SBFC/CMC-coated handsheets showed reductions ranging from 1.2 to 1.7 log_10_ compared to control samples, corresponding to an 84–89% reduction. This places the coating within the performance range reported in the literature for natural polymer-based antibacterial films^38,39^

### 3.8 Double-Sided-Coated Handsheets—Antibacterial Performance

The control handsheet was coated with a 50:50 (vol/vol) SBFC/CMC solution and dried, the sheet was flipped, and the same procedure was repeated. The optical density (OD600) of E. coli was adjusted to 0.143 using a UV–vis spectrophotometer. The bacterial colony count for the control E. coli sample was 13, while the untreated control handsheet had more than 19 colonies, indicating an increase of over 46% in bacterial growth. The double-sided-coated handsheet (50:50 vol: vol SBFC/CMC) showed a significantly lower bacterial count of just 2, with an 84.6% reduction compared to the control E. coli sample and at least a 89.5% reduction compared to the untreated control handsheet (Figure 23).

**Figure 23:**
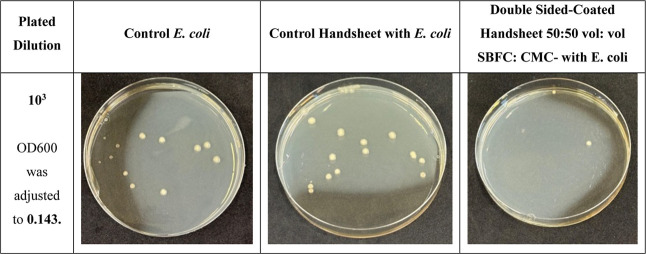
Antibacterial test results with double-sided-coated handsheet 50:50 vol: vol SBFC/CMC, with E. coli.

### 3.9 Antibacterial Test—New Sheet Coated with SBFC Only

Another trial of the antibacterial effect on E. coli was analyzed by newly coated sheets treated with only the SBFC solution. The optical density (OD600) of the bacterial suspension was adjusted to 0.166 by using a UV–vis spectrophotometer. The bacterial colony count for the control E. coli sample was 11; the untreated control sheet had 17 colonies, showing a 54.5% increase in bacterial growth compared to the control E. coli sample. The SBFC-coated sheet showed complete antibacterial activity with zero bacterial colonies, resulting in a 100% reduction compared to both the control E. coli sample and the untreated sheet (Figure 24).

**Figure 24:**
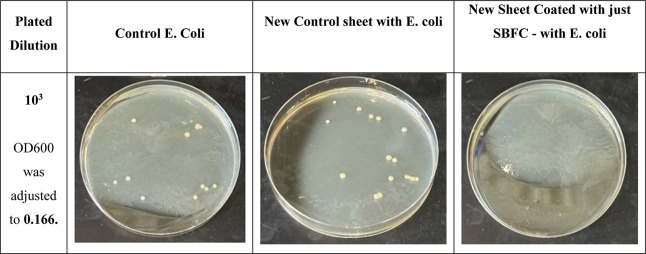
Antibacterial test results with the new sheet coated with just SBFC—with E. coli.

## 4 Conclusion

The study focused on improving the mechanical and antibacterial properties of the paper. The formulation was optimized by using a citric acid modification process for soybean flour, resulting in an increase in carboxyl content. Final optimized conditions were 140 °C for 2 h with 100% citric acid and 15% Na_2_HPO_4_. FTIR spectra proved successful esterification in citric acid-modified soybean flour, with a new ester carbonyl peak at 1711 cm^–1^. Citric acid-modified soybean flour was further complexed with chitosan. Further, a 50:50 vol: vol SBFC/CMC solution was optimized and used as a coating material for handsheets.

Surface morphology analysis through SEM confirmed that the coating effectively filled gaps between fibers, creating a denser structure compared with the control handsheets. Tensile strength testing, following TAPPI T494 standards, showed that the coated handsheets had a significant improvement in tensile strength (1.552 kgf) compared to the control handsheets (0.959 kgf).

Both antibacterial treatments, SBFC-only coating and SBFC/CMC (50:50 vol: vol) double-sided coating, showed significant bacterial reduction. The SBFC-only coated new sheet showed complete antibacterial activity, resulting in a 100% reduction in bacterial colonies, whereas the double-sided 50:50 vol: vol SBFC/CMC-coated handsheet showed an 84.6% reduction compared to the control E. coli sample and at least an 89.5% reduction compared to the untreated handsheet. Additionally, antibacterial testing at OD600 values of 0.104, 0.138, and 0.157 showed consistent reductions in bacterial colony counts across 3 trials, with the coated handsheet reducing growth by up to 80% compared to control E. coli. These results suggest that the SBFC-only coating is more effective in eliminating bacterial colonies entirely; the SBFC/CMC coating still provides substantial bacterial reduction and also improves the mechanical strength of the handsheet. If antibacterial efficiency is the priority, the SBFC-only coating is superior due to its complete bacterial inhibition. However, if a balance between antibacterial action and mechanical strength is needed, the SBFC/CMC coating remains a strong alternative, offering high bacterial reduction along with good strength.

While the CFU plate count method used here aligns with accepted standards such as ISO 22196, we acknowledge its limitations, including the inability to detect viable but nonculturable (VBNC) cells. Future work should incorporate complementary techniques, such as Live/Dead fluorescence staining or SEM-based membrane integrity assessment, to confirm bactericidal activity.
